# FHC, an NS4B-interacting Protein, Enhances Classical Swine Fever Virus Propagation and Acts Positively in Viral Anti-apoptosis

**DOI:** 10.1038/s41598-018-26777-8

**Published:** 2018-05-29

**Authors:** Gui Qian, Huifang Lv, Jihui Lin, Xiaomeng Li, Qizhuang Lv, Tao Wang, Jing Zhang, Wang Dong, Kangkang Guo, Yanming Zhang

**Affiliations:** 1 College of Veterinary Medicine, Northwest A&F University, No. 22 Xinong Road, Yangling, 712100 Shaanxi China; 2 College of Biology & Pharmacy, Yulin Normal University, No. 1303 Jiaoyu East Road, Yulin, 537000 Guangxi China

## Abstract

Classical swine fever virus (CSFV), the etiological agent of classical swine fever, causes enormous economic loss to the pig industry. Ferritin heavy chain (FHC) is a notable anti-apoptotic protein, and existing evidence suggests that CSFV cannot induce apoptosis of host cells, however, the role of FHC in CSFV replication remains unclear. In the present study, we found that recombinant lentivirus-mediated knockdown or overexpression of FHC inhibited or enhanced CSFV replication, respectively, indicating a positive role for FHC in CSFV proliferation. Furthermore, interaction between the CSFV NS4B protein and FHC was confirmed by glutathione *S*-transferase (GST) pull-down, co-immunoprecipitation (co-IP) and confocal imaging assays. In addition, both CSFV replication and NS4B expression upregulated expression of FHC, which counteracts apoptosis by modulating cellular reactive oxygen species (ROS). These results suggest that FHC, an NS4B-interacting protein, enhances CSFV replication and has a positive role in viral anti-apoptosis by regulating ROS accumulation. This work may provide a new perspective for understanding the mechanism of CSFV pathogenesis.

## Introduction

Classical swine fever virus (CSFV), the etiological agent of classical swine fever (CSF), belongs to the genus *Pestivirus* within the family *Flaviviridae*^1^. The 12.3-kb genome of CSFV is a single positive-stranded RNA that harbors a large open reading frame (ORF) encoding a polyprotein of 3898 amino acids^2^. The polyprotein is co- and post-translationally processed into twelve proteins (C, E^rns^, E1, E2, N^pro^, p7, NS2, NS3, NS4A, NS4B, NS5A, NS5B) by cellular and viral proteases^3^.

The CSFV NS4B protein, which has a function similar to that of other flavivirus NS4B proteins, is an intracellular-membrane-associated viral replicase, that is essential to the virus life cycle^4–6^. Mutation of critical residues within the NS4B Toll/interleukin-1 receptor (TIR)-like domain leads to an attenuated phenotype and reduces the replication efficiency of the highly virulent Brescia strain. Furthermore, mutation in the NS4B NTPase motif results in no infectious virus or virus replication capability impairment^7,8^. However, a chimeric low-virulence GPE-derived virus carrying the complete Eystrup NS4B sequence showed enhanced pathogenicity and high replication efficiency^9^. In addition, the findings of our previous study indicated that an NS4B-related complex, the formation of which is facilitated by Rab5, is thought to be the CSFV replication complex^10^. Although many studies have focused on the roles of CSFV NS4B in viral virulence and genomic replication, few studies have investigated cellular NS4B-interacting proteins and their roles in CSFV replication. Additionally, there are no studies to date that provide evidence suggesting that FHC interacts with CSFV NS4B.

FHC is an anti-apoptotic protein that converts Fe[II] to Fe[III] and thereby decreases the reactive oxygen species (ROS) generated from the Fenton reaction^11–13^. Increased levels of FHC prevent the cytotoxicity of ROS, whereas decreases in FHC sensitize cells to pro-oxidant cytotoxicity^14,15^. FHC has also been reported to be upregulated by the nuclear factor kappa B (NF*-*κB) signaling pathway, antagonizing tumor necrosis factor alpha (TNF-α)-mediated apoptosis by suppressing cellular ROS^16,17^. Furthermore, FHC participates in viral infection of PK-15 cells, and the ORF4 protein of porcine circovirus type 2 antagonizes apoptosis by stabilizing the level of FHC through physical interaction^18^. As CSFV is able to delay apoptosis by downregulating ROS-dependent RIG-I-like receptor (RLR) signaling, thus contributing to persistent viral infection of host cells^19^, the anti-apoptotic effect of FHC may also be involved in CSFV replication. Therefore, it is necessary to further investigate whether and how the FHC protein is involved in the CSFV life cycle and to reveal the anti-apoptotic effect of FHC on CSFV infection.

In the present study, we found that FHC promotes CSFV replication and interacts with the CSFV NS4B protein. In addition, CSFV replication and NS4B expression inhibited ROS-mediated apoptosis via upregulation of FHC levels.

## Results

### FHC interacts with NS4B

**Figure 1:**
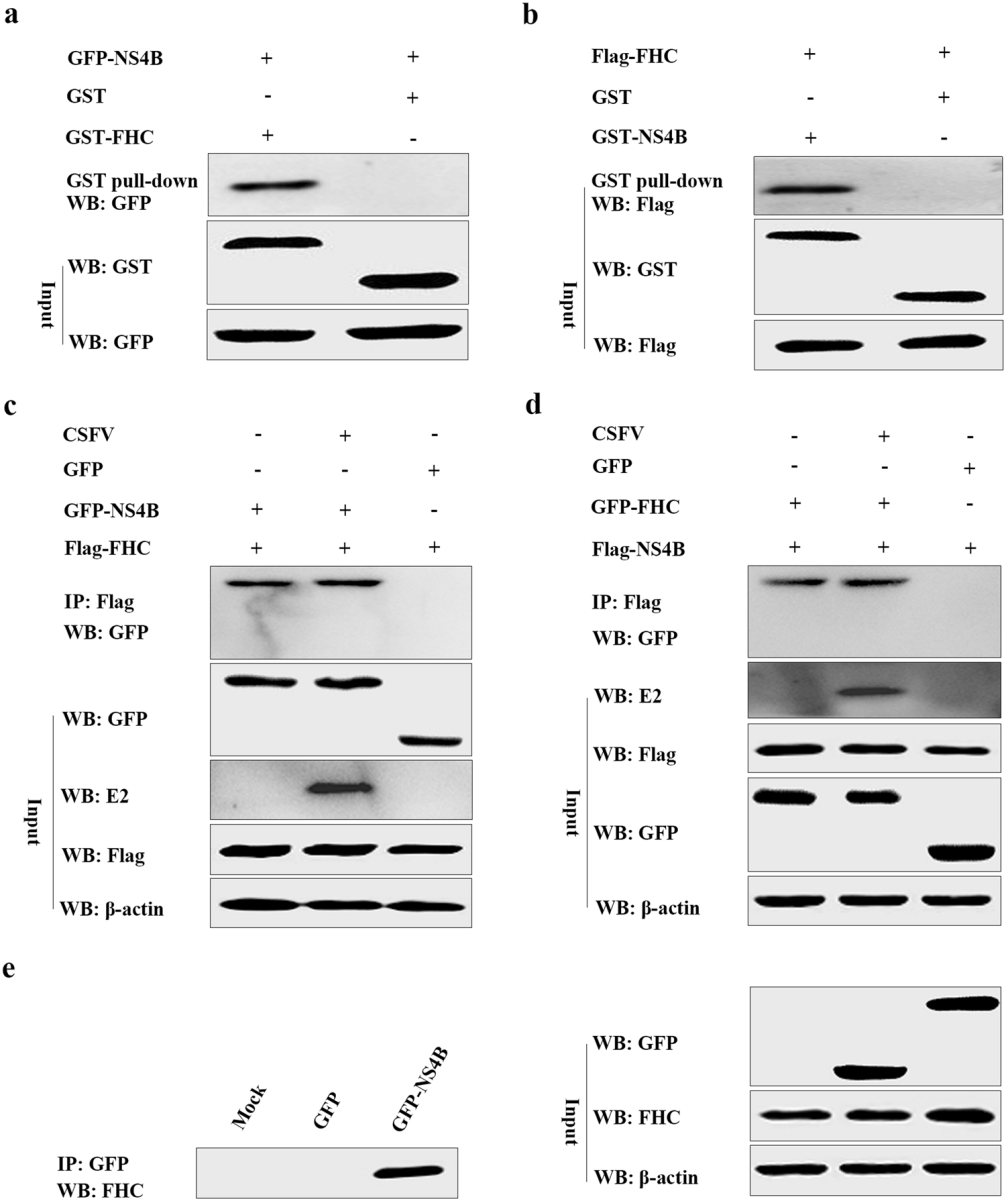
CSFV NS4B interacts with FHC. (**a**) GST pull-down assays. GST or GST-FHC fusion protein generated by *E*. *coli* BL21 (DE3) cells was purified by glutathione agarose resin, followed by incubation of the resin with GFP-NS4B protein expressed in HEK293T cells. After washing with PBS, the bound proteins were analyzed by Western blotting with anti-GFP antibodies. (**b**) Reciprocal GST pull-down was conducted as described above. GST or GST-NS4B fusion protein was conjugated with glutathione agarose resin and co-cultured with lysates containing Flag-FHC protein. After washing, the eluted proteins were analyzed by Western blotting with anti-Flag antibodies. (**c**–**e**) Co-IP assay analysis of the interaction between CSFV NS4B and FHC. (**c**) Cells were co-transfected with pEGFP-NS4B and LV-Flag-FHC (infected or not with CSFV); co-transfection of pEGFP-C1 and LV-Flag-FHC served as negative controls. A quarter of the lysates were subjected to input assays to assess β-actin, Flag-FHC, GFP, E2 and GFP-NS4B protein levels, and the remainder was subjected to IP assays. The bound proteins were analyzed by Western blotting with anti-GFP antibodies. (**d**) The reciprocal co-IP assay was conducted as described above, whereby LV-Flag-FHC was replaced with LV-Flag-NS4B and pEGFP-NS4B with pEGFP-FHC (with or without CSFV), respectively. (**e**) Co-IP analysis of the interaction between endogenous FHC and GFP-NS4B in PK-15 cells. Cells were transfected with pEGFP-NS4B, pEGFP-C1-transfected cells and mock-transfected cells served as negative controls. A quarter of the cell extract was used for input assays, and the remainder was used for IP assays. The bound proteins were analyzed by Western blotting with anti-FHC polyclonal antibodies. Full-length blots are presented in Supplementary Figure S1and 2.

It’s reported that CSFV-induced autophagy delays apoptosis and thus contributes to virus persistent infection in host cells^19^. However, there are few reports about the relation between apoptosis and CSFV replication. Remarkably, we verified FHC as a potential NS4B-interacting protein using the yeast two-hybrid assay (the data of yeast two-hybrid assay is included in a manuscript entitled “Classical swine fever virus non-structural protein 4B binds tank-binding kinase 1”, which is submitted to another journal for publication). Thus, we conducted glutathione *S*-transferase (GST) pull-down assays to verify this interaction. GST or GST-FHC proteins purified from bacteria transformed with the pGEX-6P-1 or pGEX-GST-FHC plasmid, respectively, were bound to glutathione agarose, and GFP-NS4B was added to the mixture. Western blotting using an anti-GFP antibody was performed to examine the precipitates. As shown in Fig. 1(a), GFP-NS4B was precipitated by GST-FHC not by GST alone. Reciprocal pull-down assays also showed that Flag-FHC was precipitated by GST-NS4B but not by GST alone (Fig. 1b). These results suggest that FHC binds to NS4B.

To further confirm interaction between FHC and NS4B, lysates from PK-15 cells co-transfected to express Flag-FHC and GFP-NS4B as well as Flag-FHC and GFP were incubated with Flag-agarose beads and analyzed by Western blotting using Anti-Flag M2 Affinity Gel. As shown in Fig. 1(c), GFP-NS4B was successfully precipitated by Flag-FHC, whereas negative control GFP was not. The results of reciprocal assays indicated that Flag-NS4B was able to bind to GFP-FHC but not bind to GFP (Fig. 1d). Considering the single NS4B protein of CSFV may be dysfunctional, PK-15 cells co-transfected with LV-Flag-FHC/pEGFP-NS4B or LV-Flag-NS4B/pEGFP-FHC were infected with 1.0 MOI CSFV. The results shown in Fig. 1c,d, lane 2 were consistent with those mentioned above with no CSFV infection (Fig. 1c,d, lane 1). Furthermore, lysates of cells expressing GFP-NS4B or GFP were applied to detect endogenous FHC, and the immunoprecipitation of GFP-NS4B-FHC further supported that CSFV NS4B interacts with FHC (Fig. 1e).

### FHC co-localizes with CSFV NS4B

**Figure 2:**
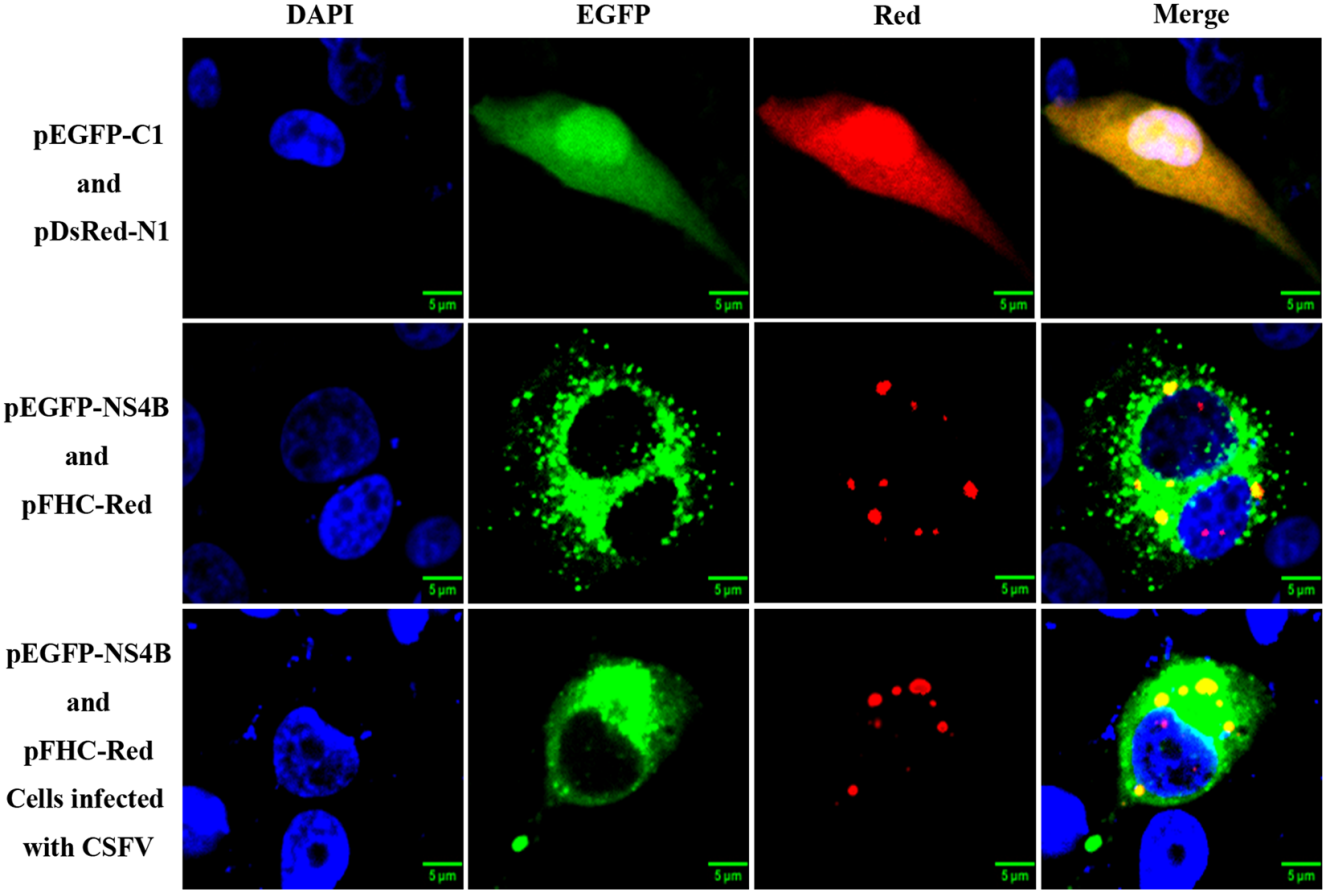
NS4B co-localizes with FHC. PK-15 cells were co-transfected with pEGFP-NS4B and pFHC-Red as well as pEGFP-C1and pDsRed-N1. Cells were fixed at 48 hpt and nuclei were visualized by staining with DAPI. The GFP-NS4B fusion protein was shown to co-localize with the FHC-Red protein in both CSFV-infected and -uninfected cells.

Because FHC was found to bind to NS4B, we next addressed whether FHC co-localizes with NS4B in PK-15 cells by co-transfecting pEGFP-NS4B and pFHC-Red as well as pEGFP-C1 and pDsRed-N1 into PK-15 cells. The transfected cells were observed by laser confocal microscopy. The yellow dot signals indicated that the fused FHC-Red protein co-localizes with GFP-NS4B in the cytoplasm of PK-15 cells whether CSFV infection occurred or not (Fig. 2). However, the co-localization coefficient of CSFV-infected cells was 0.33, larger than that of CSFV-uninfected cells (0.24), which may due to the CSFV polyprotein. Furthermore, due to the dot-like expression panel of FHC^18,20,21^, the co-localization coefficient seemed lower than general, but the co-localization phenomenon was compelling as shown in Fig. 2 array 3 lane 4. Together with the results shown in Fig. 1, these data indicate that FHC indeed interacts with CSFV NS4B.

### Knockdown of FHC inhibits CSFV replication

**Figure 3:**
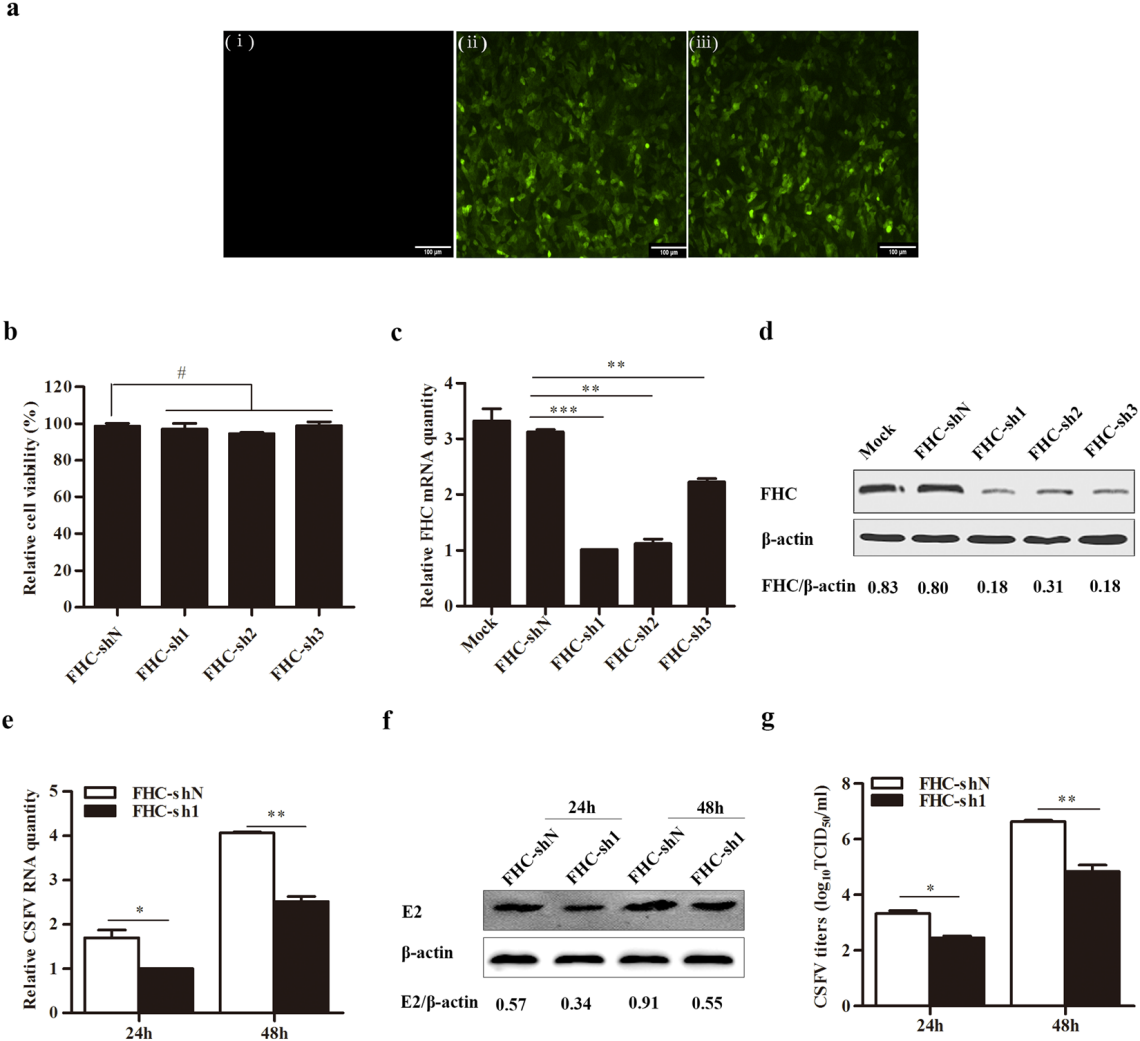
Knockdown of FHC decreases CSFV propagation. (**a**–**d**) Knockdown of FHC in PK-15 cells by shRNA. (**a**) Confirmation of lentivirus infection with the GFP reporter expressed in PK-15 cells by fluorescence detection (×100). (i) Mock-infected PK-15 cells. (ii) PK-15 cells infected with lentiviruses expressing shN. (iii) PK-15 cells infected with FHC-knockdown lentiviruses. (**b**) PK-15 cells were treated with lentivirus-based shRNA to knockdown FHC; 50 μl MTT was added to each well and incubated at 37 °C for 4 h, followed by absorbance measurement at 570 nm using a microplate reader. The mean relative cell viability of three separate experiments is shown. (**c**) RT-qPCR analysis of FHC mRNA levels in FHC-knockdown cells. (**d**) Western blotting and densitometry for FHC protein levels in FHC-knockdown cells. (**e**–**g**) Analysis of CSFV growth rates in FHC-sh1 and FHC-shN cell lines. (**e**) RT-qPCR analysis of CSFV RNA levels in FHC-sh1 and FHC-shN cell lines. (**f**) Western blotting and densitometry for E2 protein levels in FHC-sh1 and FHC-shN cells. (**g**) The titers of progeny CSFV in FHC-sh1 and FHC-shN cell lines were detected by IFA, and the average data are shown. Relative FHC mRNA and CSFV RNA levels were analyzed by RT-qPCR and normalized to β-actin levels. FHC protein expression was analyzed by Western blotting and densitometry, the densitometric FHC/β-actin or E2/β-actin ratios are shown under the blots. All RT-qPCR and IFA assays were carried out in triplicate. Full-length blots (**d** and **f**) are presented in Supplementary Figure S3. The results are shown as the mean ± SD (n = 3). *P < 0.05; **P < 0.01; ***P < 0.001; #, not significant (P > 0.05).

To determine whether FHC participates in CSFV replication, cell lines with stable short hairpin RNA (shRNA)-mediated knockdown of FHC were constructed (FHC-sh1, FHC-sh2, FHC-sh3) utilizing a recombinant lentivirus; a non-targeting FHC-shN cell line was constructed as a negative control. Cell viability upon silencing was assessed by the MTT (3-(4,5-dimethylthiazol-2-yl)-2,5-diphenyltetrazolium bromide) assay according to the manufacturer’s instructions (Nanjing Jiancheng Bioengineering Institute, China), with no lethality due to FHC knockdown observed (Fig. 3b). Among the constructs tested, FHC-sh1 exhibited the highest efficiency at inactivating FHC both at the mRNA and protein levels (Fig. 3c,d). Cells carrying FHC-sh1 or FHC-shN were then infected with CSFV at a multiplicity of infection (MOI) of 0.1. The results showed that knockdown of FHC led to lower levels of CSFV genomic RNA and CSFV E2 protein in FHC-sh1 cells, compared to the FHC-shN cells both at 24 and 48 h post-infection (hpi) (Fig. 3e,f). Additionally, knockdown of FHC significantly reduced the progeny virus titers compared with the control cells (Fig. 3g). These data indicate that knockdown of FHC suppresses the replication of CSFV.

### Overexpression of FHC enhances CSFV propagation

**Figure 4:**
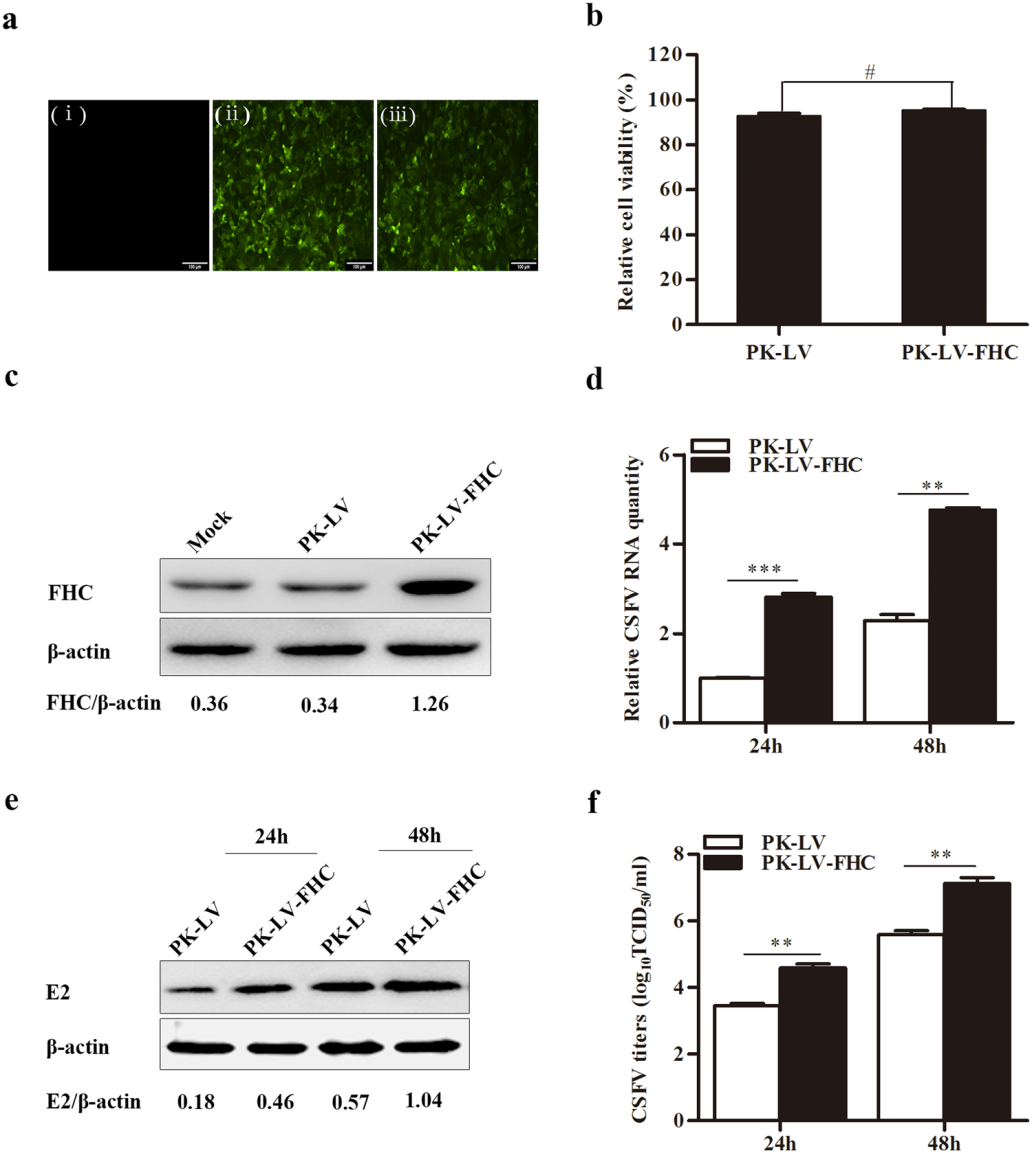
Overexpression of FHC promotes CSFV proliferation. (**a**) Fluorescence detection of the GFP reporter expressed in PK-15 cells (×100). (i) Mock PK-15 cells infected with no lentivirus. (ii) PK-15 cells infected with GFP-expressing recombinant lentiviruses. (iii) PK-15 cells infected with FHC and GFP-co-expressing recombinant lentiviruses. (**b**) PK-15 cells were treated with recombinant-lentiviruses to overexpress FHC; 50 μl MTT was added to each well and incubated at 37 °C for 4 h, followed by absorbance measurement at 570 nm using a microplate reader. The mean relative cell viability of three separate experiments is shown. (**c**) Western blotting and densitometry analysis of FHC protein levels in FHC-overexpressing cells. (**d**–**f**) CSFV growth rates in FHC-overexpressing cells. (**d**) RT-qPCR analysis of CSFV RNA levels in FHC-overexpression cells. (**e**) CSFV E2 protein levels were detected by Western blotting and densitometry. (**f**) IFA was used to determine the mean titers of progeny CSFV in FHC-overexpression cells. FHC protein expression was analyzed by Western blotting and densitometry, and the relative densitometric FHC/β-actin or E2/β-actin ratios are shown below the blots. All RT-qPCR and IFA assays were carried out in triplicate. Full-length blots (**c**,**e**) are presented in Supplementary Figure S4. The results are shown as the mean ± SD (n = 3). *P < 0.05; **P < 0.01; ***P < 0.001; #, not significant (*P* > 0.05).

To further examine the effects of FHC on CSFV replication, PK-15 cell lines stably overexpressing FHC (PK-LV-FHC) or green fluorescent protein (GFP) (PK-LV) were constructed via recombinant lentivirus infection; the MTT assay showed that recombinant lentivirus-mediated overexpression of FHC was not lethal to cells (Fig. 4b). As shown in Fig. 4(c), FHC protein levels increased notably in cells overexpressing FHC. Both PK-LV and PK-LV-FHC cells were infected with CSFV at an MOI of 0.1. As shown in Fig. 4d,e, CSFV RNA and E2 protein levels increased along with the enhanced expression of FHC, furthermore, progeny virus titers also increased markedly due to the enhanced expression of FHC both at 24 and 48 hpi, compared with the control cells (Fig. 4f). These results suggest that upregulation of FHC in PK-15 cells promotes CSFV replication.

### CSFV replication and NS4B expression upregulate expression of FHC

**Figure 5:**
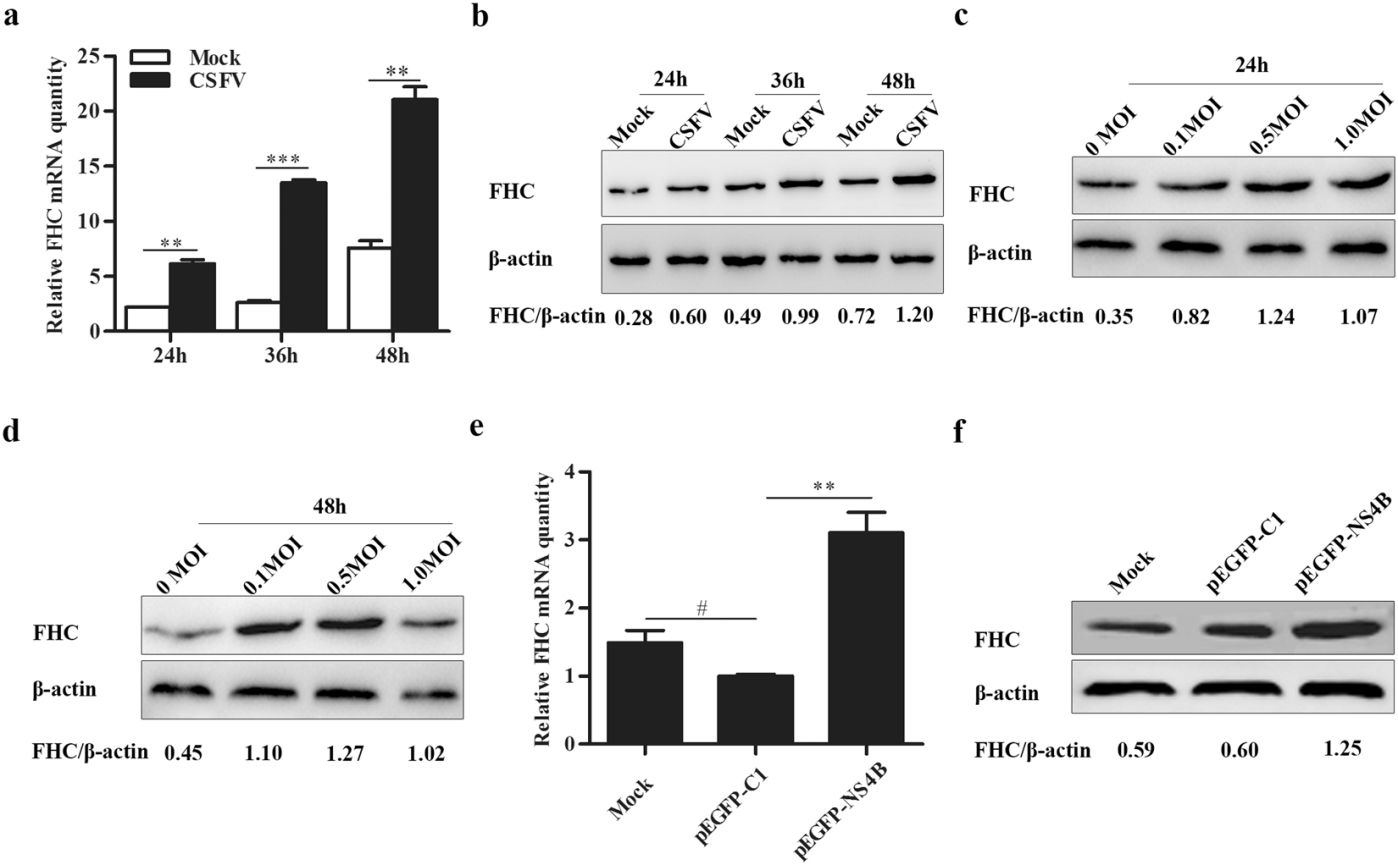
CSFV replication and NS4B expression upregulate FHC. (**a**–**e**) CSFV replication promotes FHC expression. (**a**) RT-qPCR measurement of FHC mRNA levels in CSFV-infected or -uninfected PK-15 cells. (**b**) Western blotting and densitometry analysis of FHC protein levels in CSFV infected or uninfected PK-15 cells. (**c**,**d**) PK-15 cells were infected with CSFV at various MOIs, and FHC expression was determined by Western blotting and densitometry. (**e**,**f**) NS4B expression upregulates FHC. (**e**) RT-qPCR measurement of FHC mRNA levels in PK-15 cells transfected with pEGFP-NS4B, pEGFP-C1 or mock-transfected cells, which served as controls. (**f**) Western blotting and densitometry determined FHC protein levels in PK-15 cells transfected with pEGFP-NS4B, pEGFP-C1 or mock-transfected cells, which served as controls. FHC mRNA levels were analyzed by RT-qPCR and normalized to β-actin levels. FHC protein expression was analyzed by Western blotting and densitometry, and the relative densitometric FHC/β-actin ratios are shown below the blots. All RT-qPCR assays were carried out in triplicate. Full-length blots (**b**–**d**,**f**) are presented in Supplementary Figure S5. The results are shown as the mean ± SD (n = 3). *P < 0.05; **P < 0.01; ***P < 0.001; #, not significant (*P* > 0.05).

The results presented above suggest that FHC enhances CSFV propagation, and we accordingly sought to examine the effects of NS4B expression and CSFV replication on FHC expression. PK-15 cells were infected with CSFV at an MOI of 0.1, and FHC mRNA and protein levels were assessed by RT-qPCR and Western blotting, respectively. Based on the results, both the mRNA and protein levels of FHC increased notably in CSFV-infected cells compared to CSFV-uninfected cells (Fig. 5a,b). Furthermore, to investigate whether the effects are dose dependent, PK-15 cells were infected with CSFV at MOIs of 0, 0.1, 0.5 and 1.0, and FHC protein levels were analyzed by Western blotting and densitometry at 24 and 48 hpi in comparison to levels in mock-infected cells (Fig. 5c,d). Although, CSFV infection positively modulated FHC expression, the positive effects were not strictly dose dependent.

Considering that CSFV replication promotes expression of FHC transcriptionally and translationally and that NS4B interacts with FHC, we investigated whether NS4B expression has effects similar to those of CSFV on expression of FHC. PK-15 cells were transfected with pEGFP-C1 or pEGFP-NS4B, with mock- and pEGFP-C1-transfected cells serving as negative controls. As shown in Fig. 5(e,f), the expression level of cellular FHC was the highest in pEGFP-NS4B-transfected cells compared with the negative control groups both transcriptionally and translationally, which was consistent with the results for CSFV-infected cells. These data suggest that both CSFV replication and NS4B expression increase the level of cellular FHC in PK-15 cells.

### FHC plays an anti-apoptotic role in CSFV-infected cells

**Figure 6:**
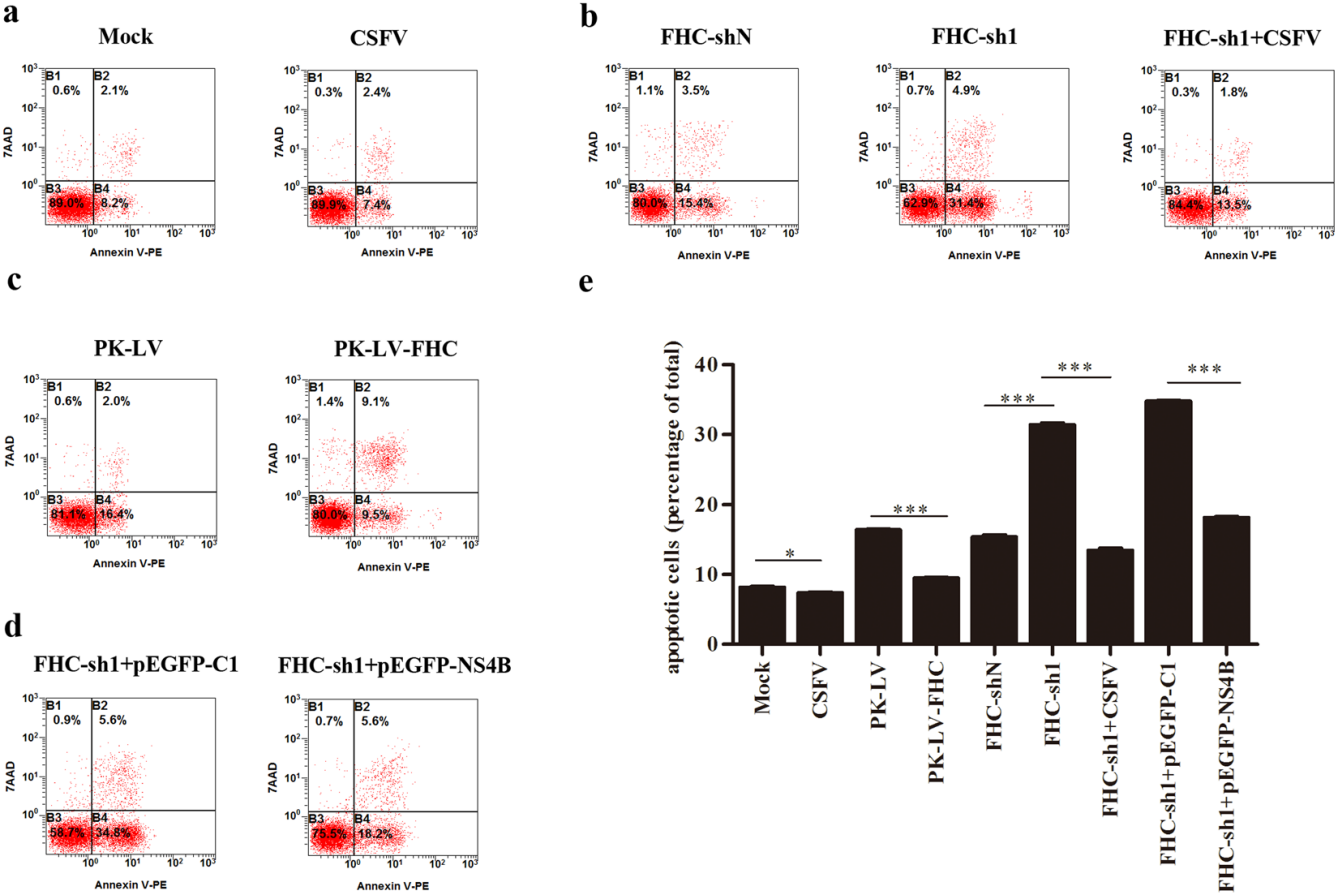
FHC plays an anti-apoptotic role in CSFV-infected cells. (**a**–**d**) Representative detection of apoptotic cells by flow cytometry. (**a**) Flow cytometry analysis of the effects of CSFV replication on apoptosis in PK-15 cells. (**b**) Apoptotic rates in FHC-sh1 cells and CSFV-infected FHC-sh1 cells. (**c**) Apoptotic rates in PK-LV-FHC and PK-LV cells. (**d**) The effects of NS4B expression on apoptosis in FHC-knockdown cells. (**e**) The mean percentage of apoptosis in three independent experiments shown in (**a**–**d**). The results are shown as the mean ± SD (n = 3). *P < 0.05; **P < 0.01; ***P < 0.001; #, not significant (*P* > 0.05).

It has been reported that FHC, the primary iron storage protein, is an anti-apoptotic protein that protects cells from apoptosis induced by a series of pro-apoptotic stimuli, including TNF-α, radiation and H_2_O_2_^16,22,23^. As CSFV cannot lead to the cytopathic effect (CPE) in cultured cells, we speculated that the anti-apoptotic role of FHC involves in the non-cytopathic of CSFV infection. To confirm the anti-apoptotic effect of FHC, stable cell lines with FHC knockdown or overexpression were constructed, and apoptotic rates were analyzed at 48 h by flow cytometry and GraphPad Prism 5.0. As shown in Fig. 6(b,c), overexpression of FHC significantly reduced the percentage of apoptotic cells compared to the control group; the rate of apoptosis in FHC-sh1 cells increased to 31.4% compared with 15.4% in FHC-shN cells, which indicates that FHC functions to counteract apoptosis. However, when FHC-sh1 cells grown on six-well plates were infected with 0.1 MOI of CSFV at 60% confluence, the apoptotic rate of FHC-sh1 cells was lower (13.5%) than that of mock-infected FHC-sh1 cells (31.4%) (Fig. 6b) at 48 hpi, similar to the results that the apoptotic rate of pEGFP-NS4B-transfected FHC-sh1 cells was 18.2%, markedly lower than that of pEGFP-C1-transfected FHC-sh1 cells (Fig. 6d). In addition, when PK-15 cells grown on six-well plates were infected with 0.1 MOI CSFV at 60% confluence, the PK-15 cells exhibited a moderated lower apoptotic rate (7.4%) than mock-infected PK-15 cells (8.2%) at 48 hpi. These data indicate that NS4B expression and CSFV replication weaken the apoptosis induced by FHC knockdown.

### NS4B and FHC limited ROS levels

**Figure 7:**
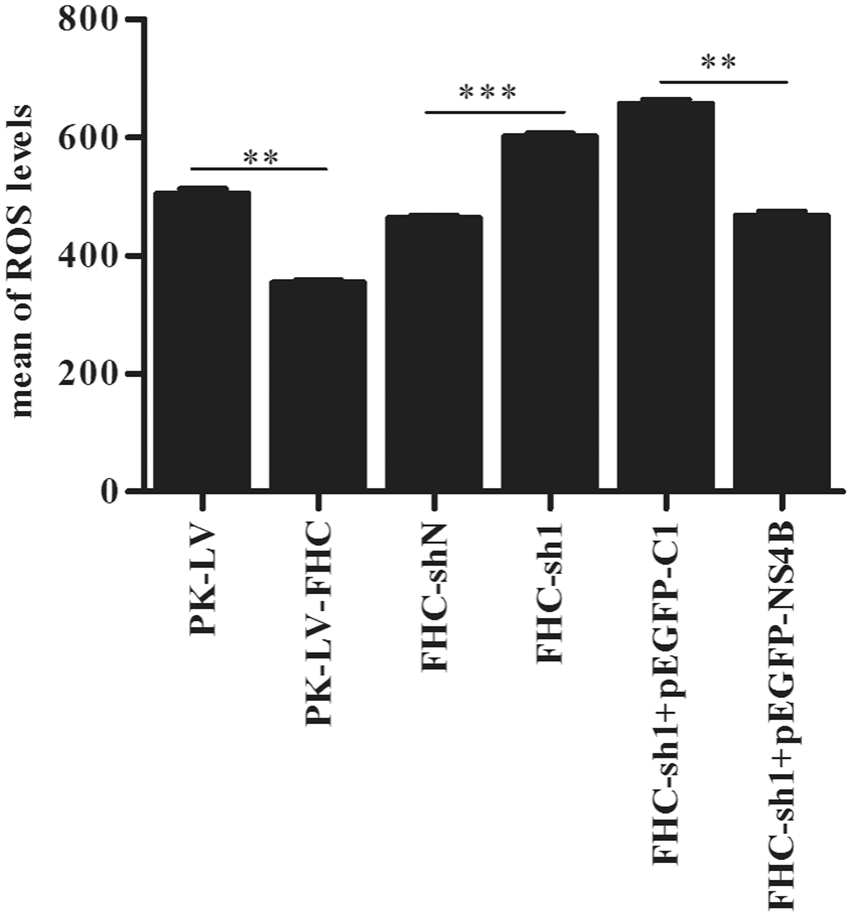
FHC limits ROS levels in PK-15 cells infected with CSFV. ROS levels were examined by flow cytometry and analyzed by FlowJo 7.6.1 Min. All flow cytometry assays were carried out in technical and biological triplicate. *P < 0.05; **P < 0.01; ***P < 0.001; #, not significant (*P* > 0.05).

ROS can mediate cell death triggered by various stimuli, including TNF-α, ceramide, radiation and chemotherapeutic agents^24–29^. Recently, CSFV was reported to sustain ROS homeostasis and to inhibit the ROS-mediated RLR signaling pathway, thus contributing to persistent CSFV replication^19^. Interestingly, FHC is an anti-apoptotic protein involved in infection by numerous viruses through regulation of ROS levels in host cells^16,18,22,23^. To investigate whether FHC and NS4B proteins influence ROS accumulation, we conducted dihydroethidium (DHE) staining and flow cytometry. As shown in Fig. 7, knockdown of FHC triggered significant ROS production, though ROS production in PK-LV-FHC cells was limited compared with that in PK-LV cells. Furthermore, ectopic NS4B expression notably decreased ROS accumulation in FHC-sh1 cells (Fig. 7). These data demonstrate that the CSFV NS4B protein limits apoptosis by maintaining cellular ROS homeostasis.

## Discussion

Host factors usually affect virus replication by interacting with the viral genome or its encoded proteins. Mitogen-activated protein kinase 2 interacts with the main CSFV antigen E2 to promote CSFV growth through attenuation of the Janus kinase/signal transducers and activators of transcription (JAK-STAT) pathway^30^. The Rab5 protein interacts with CSFV NS4B to facilitate the formation of NS4B-related complex^10^, and in Huh7 hepatoma cells, hepatitis C virus (HCV) NS5A interacts with an immunosuppressant FK506-binging protein (FKBP38) to inhibit apoptosis^30,31^. We herein report interaction between CSFV NS4B and FHC. The GST-FHC/GFP-NS4B and GST-NS4B/Flag-FHC complexes formed in GST pull-down assays suggest that CSFV NS4B binds to FHC *in vitro* (Fig. 1a,b). Visualization of GFP-NS4B and FHC-Red by laser confocal microscopy also validated that the former protein co-localizes with the latter in PK-15 cells (Fig. 2). In addition, the results of co-IP assays further confirmed that CSFV NS4B binds to FHC (Fig. 1c–e). These results indicate that CSFV NS4B interacts with the FHC protein.

The FHC protein participates in the orchestration of cellular defense against apoptosis, inflammation, stress and other host responses^17,23,32^. Moreover, FHC can ameliorate the inflammation triggered by TNF-α and protect cells from stress induced by variations in the levels of iron^17^. In addition, FHC protects hepatocytes from ischemia reperfusion injury by decreasing ROS production^22^. Our recent study suggested that the PCV2 ORF4 protein antagonizes apoptosis by stabilizing the concentration of FHC via physical interaction^18^. However, few studies have examined the effects of FHC on CSFV replication. Here, we confirm that CSFV propagation is inhibited by recombinant lentivirus-mediated knockdown of FHC (Fig. 3e–g). Furthermore, CSFV RNA levels, E2 protein levels and progeny CSFV titers increased notably in FHC-overexpressing PK-15 cells (Fig. 4d–f). These results demonstrate that FHC has a positive effect on CSFV growth.

Due to their limited genome coding capacity, proliferation of viruses cannot occur without host factors. Caveolin-1 and heat shock protein 70 have been shown to be upregulated by CSFV infection, and overexpression of both proteins promotes CSFV propagation^33,34^. Conversely, tumor necrosis factor receptor-associated factor 6 (TRAF6) and universal S10 (uS10) are downregulated by CSFV infection, and overexpression of both proteins inhibits CSFV replication^35,36^. Our results show that FHC gene and protein levels were dramatically enhanced with CSFV infection in PK-15 cells (Fig. 5a,b). Given that the anti-apoptotic effects of FHC are dose dependent^22^, we determined whether the positive effects of CSFV infection on FHC are also dose dependent by measuring FHC expression by Western blotting and densitometry. As shown in Fig. 5c,d, the FHC protein level in 1.0 MOI CSFV-infected PK-15 cells was lower than that in 0.5 MOI CSFV-infected cells but higher than that in mock-infected PK-15 cells. Two situations can lead to this phenomenon: (1) the anti-apoptotic effects of FHC are dose dependent, and when the concentration of FHC exceeds a threshold, its cytotoxicity inhibits CSFV growth; (2) excessive titers of CSFV disturb the balance between FHC and CSFV, and cells cannot produce additional FHC.

With regard to the modulation of cellular FHC expression by CSFV infection, we hypothesize that the NS4B protein, which interacts physically with FHC, can also impact FHC expression, as does CSFV. At 48 h post-transfection (hpt), both the mRNA and protein levels were the highest in pEGFP-NS4B-transfected PK-15 cells compared with negative control groups (Fig. 5e,f). Therefore, we suggest that both CSFV infection and NS4B expression enhance cellular FHC levels. It’s reported that FHC is induced downstream of NF-κB^16^, and activated NF-κB increases FHC production by binding to a *cis*-acting region located at 4.8 kb upstream of the transcriptional start site^37^. However, it has been found that CSFV infection fails to activate NF-κB both *in vitro* and *in vivo*^36,38,39^. In addition, it’s reported that CSFV-infected PK-15 cells, only exhibits a single peak of p65 activation at 1 hpi due to the treatment with TNF-α, and there is no apparent activation of p65 in CSFV-infected PK-15 cells in comparison with non-infected cells without treatment with TNF-α^40^. Therefore, we propose that the upregulation of FHC by CSFV infection and NS4B expression might not result from the activation of NF-κB. In view of the association between NS4B and FHC, the modulatory function may depend on interaction between the two proteins. Furthermore, the expression of FHC can also be regulated by cellular iron levels and oxidative stress, although the specific mechanism of this is not clear until now^41–43^. In short, more attention should be paid to elucidating the mechanism.

Apoptosis is a key pathway of host defense in viral infection and is aimed at limiting viral propagation^44^. Thus, most viruses have developed strategies to limit or delay early apoptosis for persistent infection. For example, African swine fever virus encodes a protein containing sequences homologous to B-cell lymphoma-2 (Bcl-2), and this product has been reported to inhibit apoptosis^45,46^. Furthermore, a recent study has found that HCV NS4A/B, NS5A, NS5B suppress apoptosis by blocking various members of the caspase cascade^47^. This finding coincides with others that several HCV proteins suppress apoptosis in order to promote virus persistence^48^. The CSFV genome encodes a limited number of viral proteins and it is reasonable to expect that NS4B interacts and modulates the host-cell machinery at different levels to ensure virus survival and self-proliferation. *In vitro* studies have shown that CSFV infection does not induce CPE and has no obvious influence on cell death^49^. Pei and her colleagues uncovered that CSFV infection induces autophagy and inhibits apoptosis, which leads to persistent viral infection in cell culture^19^. CSFV infection also protects aortic endothelial cells from pIpC-mediated apoptosis^50^. Moreover, FHC is a well-known anti-apoptotic protein, and we speculated that CSFV might also inhibit apoptosis by regulating expression of cellular FHC. We constructed PK-15 cell lines with FHC knockdown or overexpression via recombinant lentivirus infection and found that apoptotic rates increased significantly due to FHC knockdown; however, when FHC-sh1 cells were infected with CSFV or transfected with pEGFP-NS4B, the apoptotic rates were maintained at a level similar to that of FHC-shN (control) cells (Fig. 6). These results were further confirmed by analysis of ROS production (Fig. 7). Altogether, the results of this study indicate that the anti-apoptotic effect of FHC is involved in CSFV infection. It’s reported that CSFV-induced autophagy limits apoptosis by inhibiting both the intrinsic and extrinsic mechanisms^19^. In addition, ROS is reported to involve in both intrinsic and extrinsic apoptosis^26,27^. Therefore, CSFV might also exploit FHC to limits both intrinsic and extrinsic apoptosis, and this point will be further investigated. Nevertheless, our data provide a novel perspective that the increase in FHC levels by NS4B contributes to CSFV infection.

In summary, this study demonstrates for the first time that FHC interacts with the CSFV NS4B protein and promotes CSFV replication in PK-15 cells. Moreover, upregulation of FHC expression induced by CSFV limits PK-15 cell apoptosis via the control of ROS production. These findings may provide a new perspective for clarifying the CSFV life cycle.

## Materials and Methods

### Cells, Virus and Antibodies

Porcine kidney (PK-15) cells, swine testicular (ST) cells and human embryonic kidney (HEK293T) cells were grown in Dulbecco’s minimal essential medium (DMEM; Gibco, UK) supplemented with 10% fetal bovine serum (FBS; Gibco, UK) and 5% CO_2_ at 37 °C. CSFV (Shimen strain) was purchased from the Control Institute of Veterinary Bio-products and Pharmaceuticals (China). All the experiments related to CSFV were performed in the P3 biosafety laboratory abiding strictly by Laboratory Biosafety Manual in our lab.

A rabbit anti-FHC polyclonal antibody was purchased from Abcam (UK). Anti-E2 antibody was produced in mouse (Ab-mart, Shanghai, China). Anti-GST antibody and anti-β-actin monoclonal antibody were produced in Tianjin SungeneBiotech (China). Horseradish peroxidase (HRP)-labeled goat anti-mouse and anti-rabbit IgG were produced at Vazyme Biotechnology (China). The antibodies used for the anti-GFP (agarose-conjugated) affinity gel (CMCTAG, USA) and anti-Flag M2 Affinity Gel (SIGMA, USA) were produced in mice.

### Plasmids

Plasmids expressing GFP-NS4B or GST-NS4B were separately constructed by cloning the CSFV NS4B cDNA into vector pEGFP-C1 or pGEX-6p-1 (Clontech, USA). LV-Flag-FHC was constructed by cloning FHC into the pCDH-CMV-MCS-EF1-GreenPuro vector (SBI, Mountain View, CA, USA). To obtain pGEX-GST-FHC, pEGFP-FHC and pFHC-Red, the FHC coding sequence was cloned into pGEX-6p-1, pEGFP-C1 and pDsRed-N1, respectively. The plasmid LV-Flag-NS4B was constructed and stored in our lab. Three pairs of shRNA targeting porcine FHC and a negative control shRNA were designed and cloned into the lentivector pCDH-U6-MCS-EF1-GreenPuro (SBI, Mountain View, CA, USA) to generate FHC-sh1, FHC-sh2, FHC-sh3 and FHC-shN. All plasmids were confirmed by restriction digestion (BamH I and EcoR I) and sequencing.

### Construction of stable cell lines with FHC overexpression and knockdown

Recombinant lentiviruses overexpressing or knocked down for FHC were prepared as previously described^33^. Briefly, HEK293T cells grown in six-well culture plates were co-transfected with LV-Flag-FHC or FHC-shRNAs along with three assistant plasmids (pGag, pRev, pVSV-G, 3:1:1:1) utilizing TurboFect (Thermo, USA) according to the manufacturer’s protocol (LV and FHC-shN served as negative controls). At 48 hpt, cell culture supernatants were collected as recombinant lentiviruses by centrifugation at 25 °C, 3,000 × *g* for 5 min, and lentivirus titers were determined by 50% tissue culture infectious dose/mL (TCID_50_/ml) assay in HEK293T cells. Thereafter, PK-15 cells were infected with recombinant lentivirus at an MOI of 1.0. After culturing for 48 h, the cells were treated with puromycin (Thermo, USA) for selection. Finally, fluorescence microscopy (Nikon, Japan) and Western blotting were employed to evaluate FHC expression.

### RT-qPCR

RT-qPCR was used for quantifying relative mRNA levels of FHC and CSFV genomic RNA. Total cellular RNA was extracted by TRIzol (Invitrogen, USA), and first-strand cDNA was synthesized using a PrimeScript RT reagent kit with gDNA Eraser (Vazyme, China). RT-qPCR was performed with GoTaq® Master Mix (Promega, USA) according to the manufacturer’s protocol. RT-qPCR was carried out in technological and biological triplicate, and the expression levels of the target gene were normalized to β-actin. The specific primers used for RT-qPCR are listed in Table 1, and the relative expression level of genes was analyzed using the ΔΔCt method^51^.

**Table 1:** Primers in this research.

primers	sequence(5′−3′)	purpose
GST-FHC-F	CGGGATCC ATGACGACCTCGTGCTCCTC	Amplification of GST-FHC
GST-FHC-R	GGAATTCTTA GCTCTCACTGCTCCCCAGG	
GFP-FHC-F	CGGAATTCTATGACGACCTCGTGCTCCTCG	Amplification of GFP-FHC
GFP-FHC-R	CGGGATCCTTAGCTCTCACTGCTCCCCAGGGTG	
FHC-Red-F	GGAATTC ATGACGACCTCGTGCTCCTC	Amplification of FHC-Red
FHC-Red-R	CGGGATCCCGGCTCTCACTGCTCCCCAGG	
Flag-FHC-F	GGAATTCCATGGATTACAAGGATGACGACGATAAGATGACGACCTCGTGCTCCTC	Amplification of Flag-FHC
Flag-FHC-R	CGGGATCCCGCTCTCACTGCTCCCCAGG	
GST-NS4B-F	CGGGATCCTATGGCTCAGGGGGATGTGC	Amplification of GST-NS4B
GST-NS4B-R	GGAATTCTTATAGCTGGCGGATCTTTCC	
GFP-NS4B-F	GGAATTCTATGGCTCAGGGGGATGTGC	Amplification of GFP-NS4B
GFP-NS4B-R	CGGGATCCTTATAGCTGGCGGATCTTTCC	
FHC-sh1-F	GATCCGCAGGTGAAAGCCATCAAAGACAAGAGTCTTTGATGGCTTTCACCTGCTTTTTG	knockdown of FHC
FHC-sh1-R	AATTCAAAAAGCAGGTGAAAGCCATCAAAGACTCTTGTCTTTGATGGCTTTCACCTGCG	
FHC-sh2-F	GATCCGCTGGAACTGCACAAACTGGCCAAGAGGCCAGTTTGTGCAGTTCCAGCTTTTTG	knockdown of FHC
FHC-sh2-R	AATTCAAAAAGCTGGAACTGCACAAACTGGCCTCTTGGCCAGTTTGTGCAGTTCCAGCG	
FHC-sh3-F	GATCCGGATATCATGAAACCGGAGCGCAAGAGCGCTCCGGTTTCATGATATCCTTTTTG	knockdown of FHC
FHC-sh3-R	AATTCAAAAAGGATATCATGAAACCGGAGCGCTCTTGCGCTCCGGTTTCATGATATCCG	
shN-F	GATCCGCTTAAACGCATAGTAGGACTTCAAGAGAGTCCTACTATGCGTTTAAGCTTTTTG	negative control of knockdown
shN-R	AATTCAAAAAGCTTAAACGCATAGTAGGACTCTCTTGAAGTCCTACTATGCGTTTAAGCG	
FHC-F	CCGCGATGATGTGGCTTTG	qPCR for detection of FHC
FHC-R	GGTTTCATGATATCCTGAAGG	
β-actin-F	CAAGGACCTCTACGCCAACAC	qPCR for detection of β-actin
β-actin-R	TGGAGGCGCGATGATCTT	
CSFV-F	GATCCTCATACTGCCCACTTAC	qPCR for detection of CSFV
CSFV-R	GTATACCCCTTCACCAGCTTG	

Underline show restriction enzyme sites or loop ring, bolds show Flag tag.

### Western blotting

Cells grown in six-well plates were collected and lysed with radioimmunoprecipitation buffer (RIPA, Genshare, China) containing the protease inhibitor phenyl methane sulfonyl fluoride (PMSF, Beyotime, China) on ice for 30 min. 2 μl of the protein sample was quantified by a bicinchoninic acid (BCA) Protein Assay Kit (Genshare, China), and the remaining sample was boiled with 5× sodium dodecyl sulfate (SDS) sample buffer for 5 min. Equal amounts of protein were separated by 12% SDS-polyacrylamide gel electrophoresis (PAGE) and transferred onto polyvinylidene fluoride (PVDF) membranes (Millipore, USA). After blocking with 5% skim milk, the membranes were incubated with primary antibodies, followed by incubation with HRP-conjugated secondary antibodies. Immunoreactive bands were visualized by an enhanced chemiluminescence (ECL) Western blotting analysis system (Thermo, USA). The cellular protein β-actin served as an internal control.

### GST pull-down assays

GST-FHC was expressed in *E Escherichia coli* BL21 (DE3) cells and GFP-NS4B was generated in HEK293T cells. All procedures were performed according to the Pierce® GST Protein Interaction Pull-Down Kit manufacturer’s instructions (Thermo, USA). In brief, GST or GST-FHC expressed in *E*. *coli* BL21 cells was treated with pull-down lysis buffer on ice for 30 min, followed by immobilization on an equilibrated glutathione agarose resin for 2 h at 4 °C. The resin was then washed five times with wash solution (TBS: pull-down lysis buffer = 1:1). HEK293T cell cultures were treated as described above. Supernatants were added to the resin and incubated overnight at 4 °C. After washing five times, proteins were eluted with glutathione elution buffer. The eluted proteins were detected by Western blotting with anti-GFP antibody. In addition, the interaction between GST-NS4B and GFP-FHC was also verified as described above.

### Co-Immunoprecipitation (co-IP) assay

PK-15 cells grown in six-well culture plates were co-transfected with pEGFP-NS4B and LV-Flag-FHC (with or without CSFV infection) at 60% confluence; cells co-transfected with pEGFP-C1 and LV-Flag-FHC served as negative controls. At 48 hpt, the cells were washed with PBS and harvested with immunoprecipitation (IP) lysis buffer (Beyotime, China) containing the protease inhibitor PMSF. After centrifugation, a quarter of the supernatant was used for input analysis, and the remainder was prepared for IP experiments with ANTI-FLAG M2 Affinity Gel (Sigma, USA) according to the manufacturer’s instructions. In brief, 50 μl of the resin was centrifuged at 9,000 × *g* for 30 s and rinsed three times with 1 ml Tris-buffered saline (TBS; 50 mM Tris-HCl, with 150 mM NaCl, pH 7.4). The cell lysates were added to the equilibrated resin and rocked gently on a rotating platform overnight at 4 °C. The resin was washed three times with 1 ml of TBS and boiled with 5× SDS sample buffer, followed by Western blotting analysis with an anti-GFP antibody. The interaction between Flag-NS4B and GFP-FHC was also verified as described above. Furthermore, interaction between NS4B and endogenous FHC was tested and verified, with PK-15 cells grown in six-well plates being transfected with pEGFP-NS4B or pEGFP-C1. At 48 hpt, the cells were treated as described above, and after centrifugation, the supernatants were subjected to immunoprecipitation. The immune complexes were washed three times with ice-cold lysis buffer and subjected to SDS-PAGE followed by Western blotting analysis using a rabbit anti-FHC antibody.

### Confocal microscopy

PK-15 cells were cultured in laser co-focusing dishes and co-transfected with pEGFP-NS4B and pFHC-Red (with or without CSFV infection), as well as pEGFP-C1and pDsRed-N1. At 48 hpt or 36 hpi, the cells were washed three times with PBS, fixed with 4% paraformaldehyde at 25 °C for 20 min, and washed another three times with PBS. The cells were stained with 4′,6-diamidino-2-phenylindole (DAPI, Solarbio, China) at 25 °C for 10 min and washed three times with PBS. Images were captured by laser confocal scanning microscopy (Leica, Germany).

### Indirect immunofluorescence assay (IFA)

CSFV titers were determined by indirect immunofluorescence assay (IFA) as described previously^52^. Briefly, ST cells were cultured in 96-well plates; progeny viruses were diluted in DMEM supplemented with 2% FBS and added to the ST cells at 60% confluence. At 48 hpi, the cells were fixed with 1:1 stationary liquid (methanol: acetone) at 4 °C for 20 min and washed three times with PBS. The fixed cells were permeated with 1% Triton X-100 at 25 °C for 15 min. After another three washes, the cells were incubated with 5% skim milk at 37 °C for 2 h and then with anti-CSFV polyclonal antibodies overnight at 4 °C. The cells were next incubated with a fluorescein isothiocyanate (FITC)-labeled rabbit anti-pig IgG antibody (Sigma, USA) at 37 °C for 2 h. Immunofluorescence was observed by fluorescence microscopy (Nikon, Japan). Virus titers are expressed as TCID_50_/ml.

### Cell apoptosis detection by flow cytometry

To determine the impact of FHC on apoptosis in established cell lines, annexin V-phycoerythrin (PE) and 7-amino-actinomycin D (7AAD) (SouthernBiotech; USA) staining as well as flow cytometry were performed. Briefly, PK-15 cells grown in six-well plates were digested with trypsin and suspended to a concentration of 0.5–1.0 × 10^6^ cells ml^−1^. Subsequently, 5 μl annexin V-PE were added to 100 μl cells in a new tube, which were incubated on ice for 15 min., followed by sequentially adding 385 μl cold binding buffer and 10 μl 7AAD to each tube, which were incubated on ice for another 15 min. Apoptotic cells were examined using a Coulter Epics XL FACS (Beckman, USA).

### Detection of intracellular ROS

Dihydroethidium (DHE, Beyotime, China) staining and flow cytometry were performed to determine the impact of FHC on intracellular ROS in established cell lines. Cells grown in six-well culture dishes were incubated with 2.5 μM DHE at 37 °C for 20 min and washed three times before being treated with trypsin and harvested with DMEM without serum. ROS fluorescence was measured by flow cytometer (PARTEC, Germany) with excitation emission at 605 nm.

### Statistical analysis

Data are presented as the mean ± standard deviations (SD). Differences in each group were examined for statistical significance using Student’s *t*-test, and a *P* value less than 0.05 was considered statistically significant.

### Data availability

All data in this research are available without restriction.

## Electronic supplementary material

supplementary information
