# Supplementary material for: FHC, an NS4B-interacting Protein, Enhances Classical Swine Fever Virus Propagation and Acts Positively in Viral Anti-apoptosis
Source: Sci Rep. 2018 May 29;8:8318. doi: 10.1038/s41598-018-26777-8 (PMC5974352; doi:10.1038/s41598-018-26777-8)
Supplement: Supplementary file 1 — supplementary information [file 41598_2018_26777_MOESM1_ESM.pdf]

# FHC, an NS4B-interacting Protein, Enhances Classical Swine Fever Virus Propagation and Acts Positively in Viral Anti-apoptosis

Gui Qian[1], Huifang Lv[1], Jihui Lin[1], Xiaomeng Li[1], Qizhuang Lv[2], Tao Wang[1], Jing Zhang[1], Wang Dong[1], Kangkang Guo[1], Yanming Zhang[1,]*

[1] College of Veterinary Medicine, Northwest A&F University, No. 22 Xinong Road, Yangling, 712100, Shaanxi, China.

[2] College of Biology & Pharmacy, Yulin Normal University, No. 1303 Jiaoyu East Road, Yulin, 537000, Guangxi, China.

*Corresponding author

Yanming Zhang. Tel.: +86 2987092040

Fax: +86 2987091032

E-mail address: zhangym@nwsuaf.edu.cn

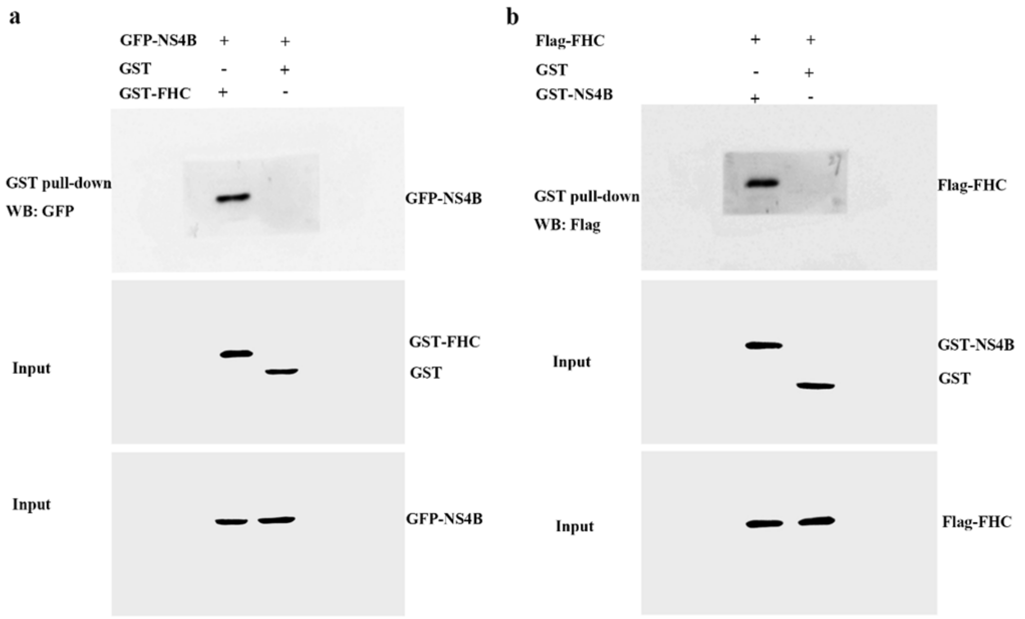


**Figure S1 Full-length blots for Figure. 1a and b.**

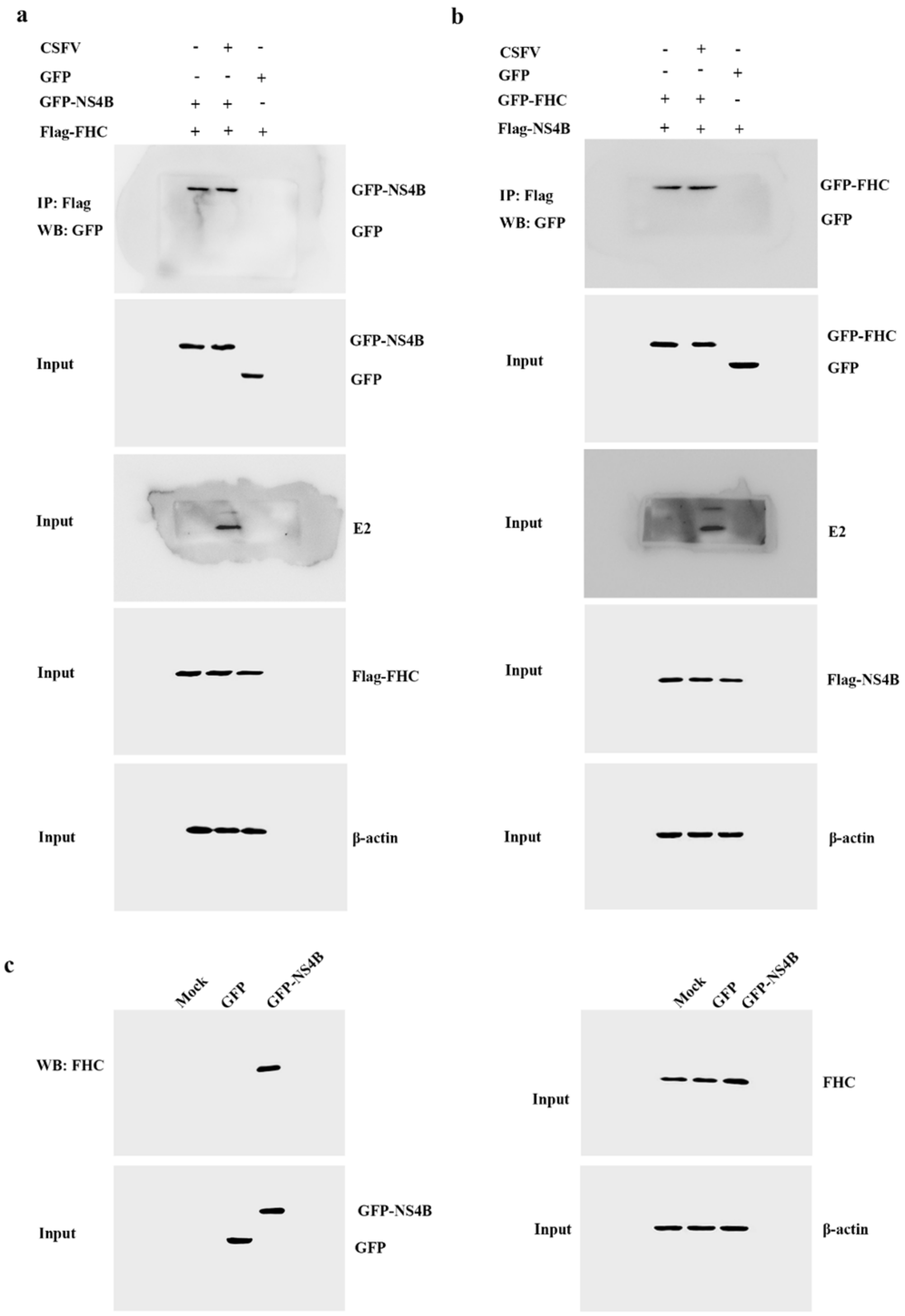


Figure. S2 Full-length blots for Figure. 1c, d and e.

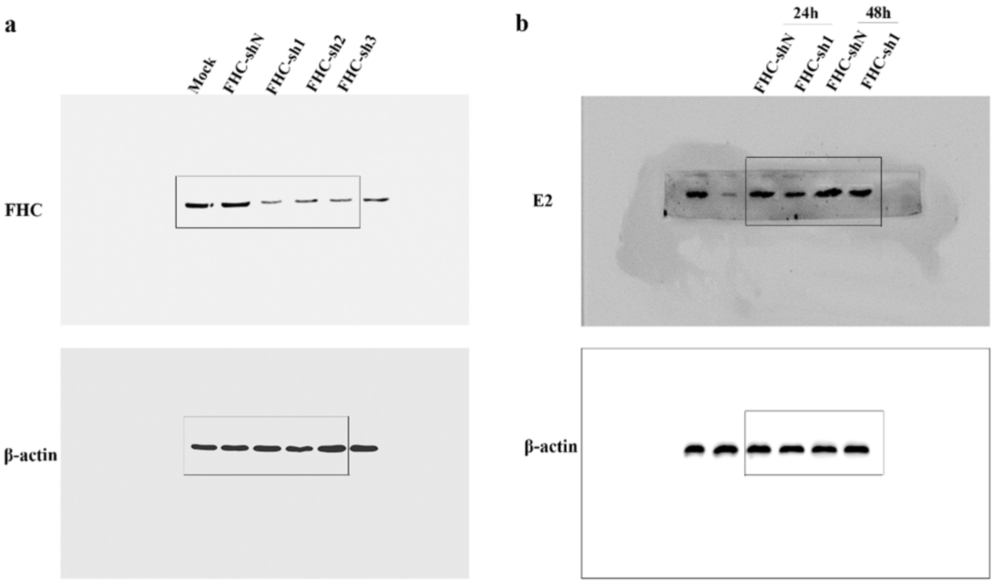


Figure. S3 Full-length blots for Figure. 3d and f.

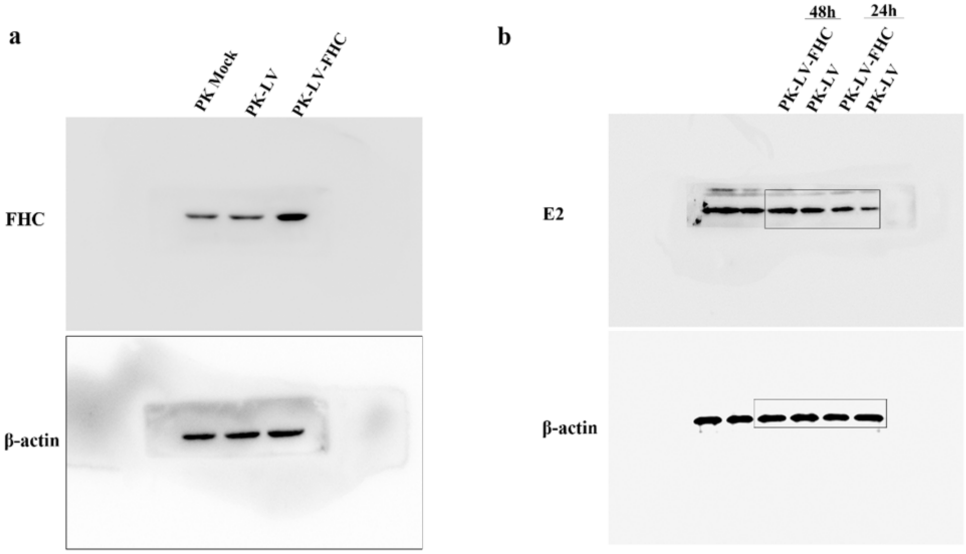


Figure S4 Full-length blots for Figure. 4c and e.

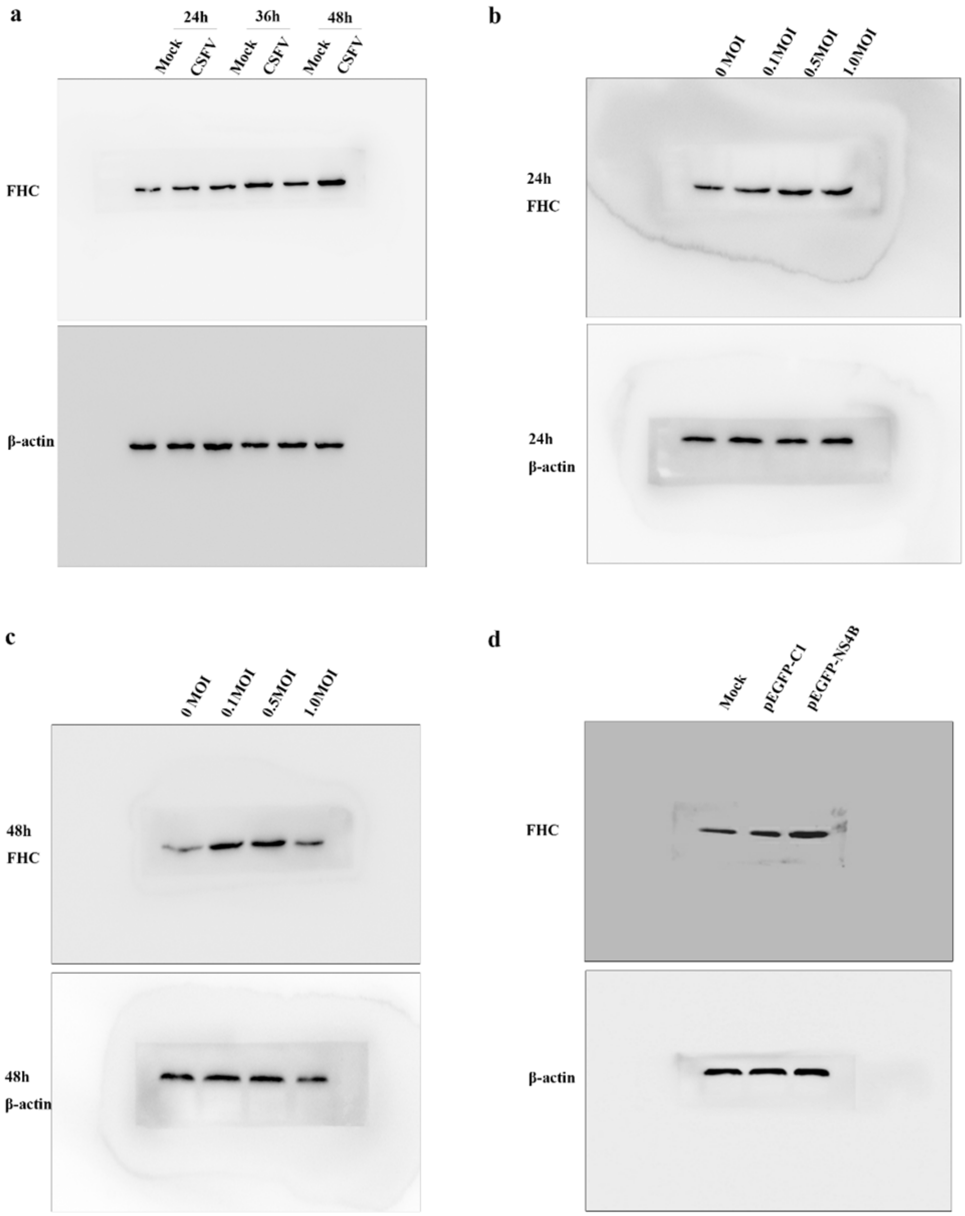


**Figure. S5 Full-length blots for Figure. 5b, c, d and f.**
